# Special edition booklet: Interprofessional Training – Published by the Robert Bosch Stiftung and the Gesellschaft für Medizinische Ausbildung

**DOI:** 10.3205/zma001037

**Published:** 2016-04-29

**Authors:** Bernadette Klapper, Christian Schirlo

**Affiliations:** 1 Robert Bosch Stiftung GmbH, Themenbereich Gesundheit, Stuttgart, Germany; 2 Universität Zürich, Medizinische Fakultät, Dekanat, Stabsstellenleiter, Zürich, Switzerland

## Editorial

Today, anyone who chooses a career in the health care sector in Germany, Austria, and Switzerland usually stays among their peers – both during their studies as well as their professional training. To date, study courses in the fields of medicine and care, health care and nursing training, and studies and training in the therapeutic professions such as physiotherapy or speech therapy, as well as the medical-technical careers, almost entirely focus on one profession. However, in light of the increase in chronic diseases and the growing importance of multiple morbidities and dementia – also in regards to demographic changes – interprofessionally coordinated care is becoming more and more important. These demographic and epidemiological changes are resulting in increasingly complex situations and processes for care, the handling of which – in terms of the health, safety, and quality of life of the patient – can only be successful through sound collaboration. An awareness of the tasks, competencies, and responsibilities of the other career fields is fundamental for collaboration in ensuring high-quality care for the patients.

However, acquisition of these interprofessional skills, abilities, and expertise, as well as knowledge of the intersections between and transition from one health care profession to another, is hardly been supported. Training in the areas of communicative and social skills in interprofessional settings with regard to successful instances of collaboration is also only found sporadically in various curricula. Collaborative learning can foster the development of these skills and combat the formation of barriers and misconceptions about the other job categories. 

Interprofessional collaboration alone will most likely not be able to solve all future problems and challenges in patient care. However, it is a necessity to be able to meet today’s requirements and to take on new challenges professionally. This includes – for all job categories – awareness of and reflection on one’s own role, tasks, and responsibilities, as well as those of the other job categories when working together. The chances of success for collaboration are much higher if it is learned during a training or study course and continuously trained and practiced via professional development. 

Exactly this was the impetus for the Robert Bosch Stiftung to launch the Operation Team – Interprofessional Training in Health Care Professions program. The objective of this support is to disrupt the “monoprofessional” training culture and to implement a structural anchor of interprofessional training opportunities of substantial scope and quality, as well as to establish interprofessional training as an integral element in the training portfolio of the health care sector. This objective is ambitious, and the path will be long and arduous. The current conditions seem to indicate that the time is now for this integration of interprofessional training elements: the very positive response to all advertisements of the Operation Team and the impressively high number of submissions are indications of this. Over the past few years, several projects have been initiated at various locations in Germany, Austria, and Switzerland, and a number of ideas for interprofessional learning and teaching have been carried out. Many different curricular measures and projects in training and professional development have already been initiated, and experience has been gathered on topics ranging from structuring the content of interprofessional training courses to various constellations of target groups and institutional connections. A significant intention – and the next logical step – was therefore to record these experiences, describing the respective projects, achieving harmony between the level of research regarding interprofessional training in Germany, Austria, and Switzerland, and making this available to all committed stakeholders and interested institutions. 

The idea for this publication arose at a networking meeting for the projects supported by the Operation Team program in November 2014. Together, the Gesellschaft für Medizinische Ausbildung (GMA) and the Robert Bosch Stiftung have developed this idea further and invited the Operation Team projects, GMA members, and other committed stakeholders to take part in a joint booklet in the form of academic contributions on the topic of interprofessional training. The response to our call was overwhelming and once again confirmed the original idea. 

We are now pleased to present the “Interprofessional Training” booklet with numerous contributions. The publication is an important building block for deepening the conversation surrounding interprofessionalism, bringing the topic more into the focus of current developments in vocational policy, campaigning among critical decisionmakers, and supporting committed stakeholders in their efforts. The publication is also intended to present an up-to-date overview of selected projects and studies in the field of interprofessionalism in the German-speaking world. This is intended as an impulse for the necessary generation of additional scientific evidence on the effects of interprofessional curricular measures as well as on possible mechanisms and contexts [1] – Jill Thistlethwaite presents this in her scientific editorial in this booklet. A sequence presented by Scott Reeves, which is often implicitly accepted as causal, represents one possible model for the categorization of studies, especially with regard to methodological aspects [2]. This sequence comprises interprofessional training and development and their effects on the level of interprofessional collaboration or teamwork, or on the level of interprofessional care.

As the publisher, we hope that the original work, overviews, and comments presented here are met with substantial interest. In particular, we hope that the projects introduced find the necessary support so that they can be developed further, establish themselves at facilities, and act as beacons – nationally and, if possible, internationally – casting light across a wide swath. We hope that this booklet enriches the discourse, both in relation to scientific aspects as well as aspects of educational policy, and helps to uncover the existing holes and hurdles and to work on new methods and approaches to solutions. This hope also applies in terms of the orientation of educational institutions in the health care sector in Germany, Austria, and Switzerland towards embodied interprofessional culture of education as one of the significant requirements for sustainable and continuously improving patient care. 

## Acknowledgements

We thank all of the authors for their outstanding collaboration and contributions, as well as all reviewers for their tireless and consistent review process. Out thanks also goes out to all those who helped put this booklet together: Beate Herrmannsdörfer (GMA) for her energetic management of the contributions submitted and coordination of the peer-review process, Tanja Frey (Robert Bosch Stiftung) for her creative graphic design of the booklet, and Irina Cichon (Robert Bosch Stiftung) for her coordination of the overall project.

## Competing interests

The authors declare that they have no competing interests.
